# Sodium Hypochlorite-Assisted Photooxidation of Salicylic Acid: Degradation Kinetics, Formation, and Ecotoxicological Assessment of Intermediates

**DOI:** 10.3390/ijms262010063

**Published:** 2025-10-16

**Authors:** Waldemar Studziński, Alicja Gackowska

**Affiliations:** Faculty of Chemical Technology and Engineering, Bydgoszcz University of Science and Technology, 85-326 Bydgoszcz, Poland; alicja.gackowska@pbs.edu.pl

**Keywords:** SA, chlorination, transformation products, ecotoxicity, photooxidation

## Abstract

Detailed studies were conducted on the photooxidation of salicylic acid (SA) in the presence of sodium hypochlorite (NaOCl), which is important in the context of water disinfection processes. It was shown that NaOCl alone causes slow degradation of SA (<10% after 60 min), while its combination with UV radiation significantly increases the efficiency of the process, especially at pH 7.5–10 (up to 30% degradation in 60 min). Eleven chlorinated transformation products have been identified, including 2,6-dichlorophenol and 2,4,6-trichlorophenol, which are characterized by high environmental persistence (>96 days) and the ability to travel distances exceeding 4000 km. QSAR analyses and ecotoxicological tests (Microtox^®^, Daphtoxkit F^®^, *Lemna* sp.) confirmed the significant toxicity of some compounds to fish, daphnia, and algae. It was found that the post-reaction mixture after the NaOCl/UV process exhibits higher toxicity than SA photolysis alone, indicating a significant contribution of chlorinated intermediates to environmental risk. The results highlight the need to develop alternative methods for removing pharmaceuticals that minimize the formation of persistent and toxic by-products, and indicate directions for further research on their monitoring in the aquatic environment.

## 1. Introduction

Over the past twenty years, pharmaceuticals have been identified as a problematic group of pollutants. Despite their beneficial medicinal effects, these products are a cause for concern among scientists because they have been identified as pollutants in municipal wastewater, hospital and livestock wastewater [1,2,3], surface water [4,5], groundwater [6], drinking water [7], and marine ecosystems [8]. The use of medicines and cosmetics by humans, the excrement of farm animals under veterinary care, and illegal waste dumps are the main sources of pharmaceutical residues in the environment. Improper handling of expired medicines in households also contributes to their presence in the aquatic environment [9]. It has been shown that the concentration of pharmaceutical residues in raw sewage is at the mg·L^−1^ level. The problem is not only their quantity but also their diversity in terms of physical and chemical properties. The vast majority of these pollutants are characterized by poor biodegradability, high polarity, and high biological activity. Conventional wastewater treatment methods are insufficient to break them down into simple compounds. Pharmaceuticals with lipophilic properties only change location, as they accumulate in sediments rather than undergoing degradation.

Hydrophilic compounds, on the other hand, are transported to the environment along with treated wastewater. Incomplete removal of pharmaceutical residues in wastewater treatment plants has allowed them to spread in surface waters at levels of µg·L^−1^ and ng·L^−1^. Chemical compounds that are continuously released with treated wastewater in low concentrations often have the same type of physicochemical properties as other xenobiotics and cause biological effects; they are called pseudo-persistent micropollutants [10]. The systematic and continuous introduction of pharmaceutical residues and their bioactive metabolites into the environment can lead to long-term, high concentrations and cause continuous but imperceptible adverse effects on aquatic and terrestrial organisms. Research conducted by Wiese et al. showed significant concentrations of oxazepam in *Eurasian perch*, with a bioaccumulation factor of 12 [11]. In turn, Fong and co-authors observed the effect of antidepressants (venlafaxine and fluoxetine) on the behavior of two species of marine snails [12]. Furthermore, these pollutants are not inert to environmental factors and undergo further transformations, creating products with significantly higher toxicity than the substrates themselves [13,14,15]. The harmful effects of pharmaceuticals and their resistance to biological and physicochemical wastewater treatment mean that effective techniques for their removal are being sought [16,17]. Disinfection plays a particularly important role in the case of water. The microbiological safety of water is one of the basic conditions for its use for both recreational and food purposes. Due to its low cost, chlorine is the most commonly used chemical oxidant in the world for disinfecting drinking water, swimming pools, and wastewater. Chlorine is a strong oxidizing agent and has strong antimicrobial properties. It has been used for over 100 years to disinfect municipal water supplies. The commercially available forms of chlorine that are commonly used in manufacturing are sodium hypochlorite and calcium hypochlorite [18]. It is assumed that free chlorine in water occurs in the form of HOCl, Cl_2_, and Cl_2_O (reactions 1–3), with HOCl being the main chlorinating agent [19].

HOCl ⇌ H^+^ + OCl^−^ (1)

HOCl + Cl^−^ + H^+^ ⇌ Cl_2_ + H_2_O (2)

2 HOCl ⇌ Cl_2_O + H_2_O (3)

On the one hand, the forms of free chlorine present in water have a beneficial effect on the inactivation of pathogens, and it has also been shown that chlorination reduces the concentration of certain parent drugs in drinking water [20]. On the other hand, although pharmaceuticals are present in water at low concentrations, they are sufficient to initiate chemical chlorination reactions. Free forms of chlorine can react with electron-rich organic compounds to form disinfection by-products [21,22,23,24]. By-products of chlorination have been linked to adverse reproductive effects, certain types of cancer, and developmental problems [23].

In these studies, SA was used as a model example of pharmaceutical residue contaminants. SA belongs to a group of non-steroidal drugs with anti-inflammatory, analgesic, and antipyretic properties. Annual global production of the acid is approximately 180,000 Mg. SA is used for the synthesis of acetylsalicylic acid (aspirin) [25], 4-aminosalicylic acid, salicylamide, ethenzamide, phenyl salicylate, and bismuth subsalicylate. SA is a low-toxicity contaminant. Its presence in surface waters has been confirmed at a level of µg·L^−1^ [26], and in drinking water at a level of ng·L^−1^ [27]. In many studies, researchers focus on the effective elimination of SA from wastewater and the optimization of the process itself. However, it is also important to identify the by-products of the transformation and estimate their ecotoxicity, as they may pose a much greater threat to living organisms than the parent compound. Previous studies of model systems have shown that SA degrades under the influence of oxidizing agents. Depending on the process conditions, products with different concentrations and properties may be formed, which play an important role in their behavior in the environment. As a result of photooxidation of SA, in the presence of hydrogen peroxide, 2,3-dihydroxybenzoic acid, 2,5-dihydroxybenzoic acid, and pyrocatechol are formed [28]. It has been shown that these products are susceptible to the influence of hydroxyl radicals, which play a significant role in the oxidation process. As a result of the transformation of both SA and its intermediate products, compounds are formed that exhibit toxic properties [29]. In the case of free-chlorine-based disinfectants, there is a high probability of the formation of organochlorine products. Broadwater et al. showed that in chlorinated water, SA can undergo halogenation reactions, resulting in the formation of mono- and dichlorinated derivatives of the acid [19].

The key objective of this study is to identify and characterize the intermediate products formed during the photooxidation of salicylic acid in the presence of sodium hypochlorite. Beyond simple identification, the mobility, persistence, and ecotoxicological classification of these compounds were systematically investigated. This allows for a comprehensive assessment of their potential environmental risks. Importantly, the novelty of this research lies not only in confirming SA degradation under chlorination—already known in the literature—but in providing the first detailed characterization of chlorinated intermediates and their properties. By evaluating their stability, environmental transport potential, and toxicity to different groups of aquatic organisms, this study addresses a critical knowledge gap. Previous studies have largely focused on the efficiency of SA removal, neglecting the chemical fate of by-products. This research, therefore, contributes new insight into the environmental implications of chlorination, offering both a mechanistic understanding and a framework for risk assessment.

In the long term, the findings presented here advance our understanding of the complete transformation cycle of SA under realistic chlorination conditions, as applied in drinking water treatment. They also highlight the environmental hazards associated with secondary products, which may persist and accumulate in aquatic ecosystems. By shedding light on these overlooked processes, this study provides essential knowledge for the improved design of water disinfection practices—minimizing the formation of harmful by-products while maintaining microbial safety.

## 2. Results and Discussion

### 2.1. The Effect of NaOCl on Salicylic Acid Degradation

SA degrades slightly under the influence of NaOCl alone. This process is very slow, as after 60 min the degradation of SA did not exceed 10%. The low effectiveness of SA degradation under the influence of hypochlorite alone may be due to the fact that under the influence of hypochlorite, the pH of the solution shifts towards alkaline, in which the dominant form is OCl^−^, which is a weak oxidant with an oxidation potential of +0.89 V [30].

**Figure 1:**
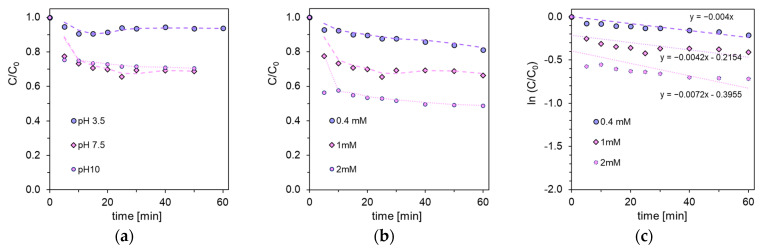
Photodegradation of SA with sodium hypochlorite depending on the solution pH (**a**). Influence of sodium hypochlorite concentration on the stability of SA (**b**). Kinetic lines of SA photodegradation with different concentrations of sodium hypochlorite (**c**).

Better results were obtained when the classic chemical oxidation process was additionally supported by the presence of UV radiation. However, in this case, the rate of SA degradation depends on the pH of the system (Figure 1a). After 60 min, the substrate concentration decreased by 30% at pH 7.5 and 10. The reaction was significantly slower at pH 3.5, with a degradation of 12% observed. In an aqueous solution, sodium hypochlorite dissociates into two main chemical compounds: hypochlorous acid (HOCl) and hypochlorite ion (OCl^−^). The proportion of HOCl and OCl^−^ forms present in water depends primarily on the pH. Under alkaline conditions (above pH 7), the OCl^−^ ion dominates, while a pH below 7 shifts the equilibrium towards HOCl. When the pH of the sodium hypochlorite solution falls below pH 4.5, the equilibrium of the solution shifts towards the production of free Cl_2_.

When using the NaOCl/UV system, the process of photodegradation of hypochlorite occurs, resulting in the formation of reactive hydroxyl radicals with a significantly higher oxidation potential (+2.8 V) than that of HOCl and OCl^−^, as well as Cl^•^ radicals. A key stage in this process is the photolysis of the hypochlorite ion (OCl^−^), which, as a result of UV radiation absorption (λ ≈ 280–300 nm), decomposes into highly reactive oxygen radicals (^•^OH), responsible for the effective oxidation of organic compounds. This phenomenon is particularly effective at pH 7.5–10, where the concentration of OCl^−^ is highest, which promotes its photolysis and increases the efficiency of the oxidation process. In an acidic environment (pH 3.5), where the presence of OCl^−^ is negligible and non-dissociated HOCl dominates, the rate of SA degradation was significantly lower, confirming the important role of the hypochlorite ion in photoinduced reactions [31].

The radicals formed can react quickly with the aromatic ring of SA or with carboxyl and hydroxyl groups. It has been observed that the rate of SA degradation depends on the concentration of the oxidant. After applying a fivefold excess of NaOCl relative to SA, the acid concentration decreased by another 20% (Figure 1c). However, the most intense increase in the rate of SA degradation is observed in the presence of UV radiation alone. Similar chlorination effects have been observed in studies on phenolic and aromatic compounds [32,33,34,35].

### 2.2. Chloro-Organic Products of SA Photodegradation in the Presence of NaOCl

**Figure 2:**
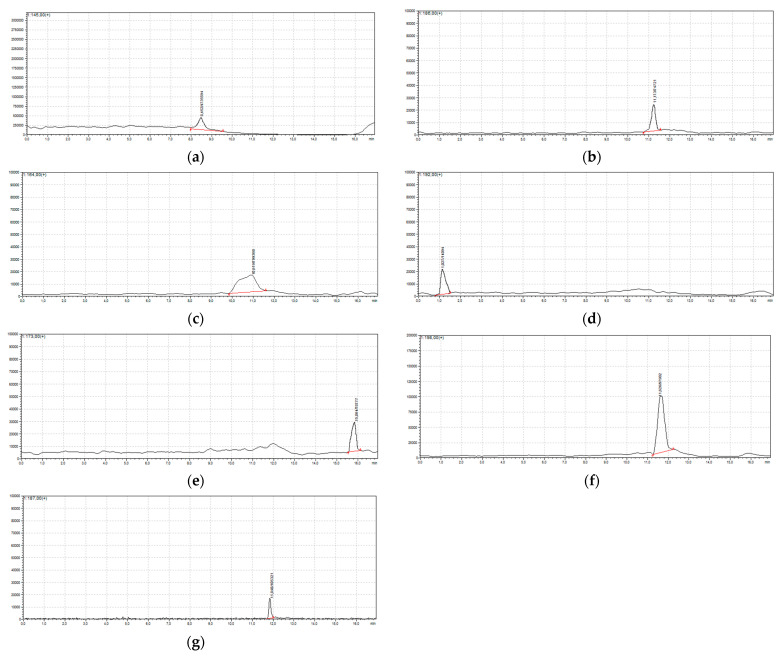
HPLC/MS chromatogram of salicylic acid (SA) solution after reaction with NaOCl/UV. Chromatographic conditions—C18 column, 3 mm × 100 mm, 2.6 µm; flow rate: 0.4 mL/min; mobile phase: water with 0.1% formic acid (A) and methanol (B); gradient elution: 95:5 *v*/*v* (0 min), 95:5 *v*/*v* (7 min), 55:45 *v*/*v* (10 min), 35:65 *v*/*v* (11 min), 5:95 *v*/*v* (12–14 min), 95:5 *v*/*v* (15–17 min). Detection: ESI+, selected ion monitoring (SIM) for [M + H]^+^ = 145, 164, 173, 186, 187, 192, 198. Peak assignments: (**a**) 4-chlorobenzene-1,2-diol, (**b**) (2-chloro-4-hydroxyphenyl)(oxo)acetaldehyde, (**c**) trichloroacetic acid, (**d**) 3,5-dichloro-4-hydroxybenzaldehyde, (**e**) chloro-hydroxybenzoic acid, (**f**) 2,4,6-trichlorophenol, and (**g**) methyl 5-chlorosalicylate.

In the presence of strong oxidizing agents, hydroxylation of the aromatic ring of SA occurs, resulting in the formation of dihydroxybenzoic acids. The mechanism of these transformations and the characteristics of the resulting products are presented in the authors’ previous work [29]. During photooxidation assisted by sodium hypochlorite, the authors also observed similar oxidation products, but due to the high reactivity of the system, these products underwent chlorination and the main products of the transformation were organochlorine products, which are the subject of this article. Products of SA photodegradation in the presence of sodium hypochlorite were identified using HPLC-MS. The masses of the products were determined based on peaks corresponding to the protonated molecule [M + H]^+^ (Figure 2).

The recorded ESI+ *m*/*z* values are consistent with the results of previous studies and provided the basis for proposing the structure of the detected compounds (Table 1). Molecules with the experimental values [M + H]^+^ 145, 164, 173, 186, 187, 192, and 198 can be assigned the structures C_6_H_5_ClO_2_, C_2_HCl_3_O_2_, C_7_H_5_ClO_3_, C_8_H_6_ClO_3_, C_8_H_7_ClO_3_, C_7_H_4_Cl_2_O_2_, and C_6_H_3_Cl_3_O. The authors also identified new compounds with values [M + H]^+^ 117, 123, 165, 175, 189, and 237, which, to the authors’ knowledge, have not been described or identified to date.

**Figure 3:**
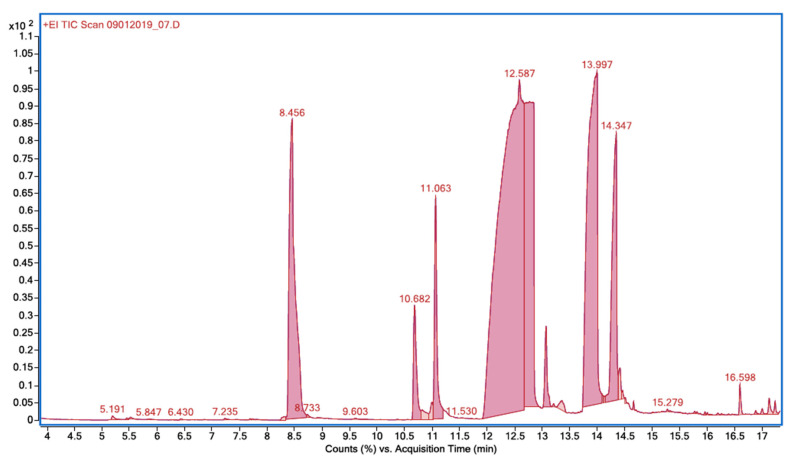
An exemplary chromatogram of the reaction mixture after photooxidation of SA in the presence of NaOCl/UV. Conditions: column ZB-5MS, (0.25 mm × 30 mm, 0.25 µm); mode SCAN *m*/*z* 50–550. The main products are listed in Table 2 according to their retention time.

GC-MS was also used to identify organochlorine compounds. For this purpose, the post-reaction solution was extracted with ethyl acetate. Figure 3 shows the corresponding chromatogram, and Table 2 lists the transformation products along with retention time and *m*/*z* values.

Most of the identified intermediates are chlorinated aromatic phenols: 2-chlorophenol; 2,6-dichlorophenol; 2,4-dichlorophenol; 5-chloro-2-hydroxybenzoic acid; 3-chloro-2-hydroxybenzoic acid; and methyl 5-chlorosalicylate. Ambauen et al. and Quintana et al. revealed similar products in the chlorination of SA: 3-chloro-2-hydroxybenzoic acid; 5-chloro-2-hydroxybenzoic acid; and 3,5-dichloro-2-hydroxybenzoic acid. DFT (Density Functional Theory) computations and natural bond orbital analysis showed that the 3- and 5-positions in the aromatic ring of SA have the highest spin density. For this reason, the ^•^OH and ^•^Cl radicals react mainly at positions 3 and 5, and to a lesser extent at positions 4 and 6 [36,37]. Indeed, intermediates of electrooxidation and chlorination of SA did not contain 2,6-dihydroxybenzoic acid and 4-chloro-2-hydroxybenzoic acid. In this study, 3-chloro-2-hydroxybenzoic acid, 5-chloro-2-hydroxybenzoic acid, and methyl 5-chlorosalicylate were detected at the initial stages of photooxidation, while chlorophenols were detected at subsequent stages. The decarboxylated products appear to be secondary intermediates of SA photodegradation, which is consistent with the report of Liu et al. [38]. The main chlorinated phenols were found to be 2-chlorophenol, 2,4-dichlorophenol, and 2,6-dichlorophenol (Table 1). Literature reports on chlorination of phenols confirm that monochlorinated products are mainly 2-chlorophenol and 4-chlorofenol. Further chlorination results in dichlorophenols and trichlorophenols, such as 2,6-dichlorophenol, 2,4-dichlorophenol, and 2,4,6-trichlorophenol [39,40,41,42]. Thus, the addition of chlorine to phenols is mainly at positions 2, 4, and 6.

Ge et al. additionally found that the concentration of phenol derivatives depends on the pH of the system. Phenol, 2-chlorophenol, and 4-chlorophenol are the dominant compounds in an acidic environment, while dichlorophenols (3-chloro-2-hydroxybenzoic acid, 5-chloro-2-hydroxybenzoic acid) are the main components in neutral and alkaline environments [41,42].

**Figure 4:**
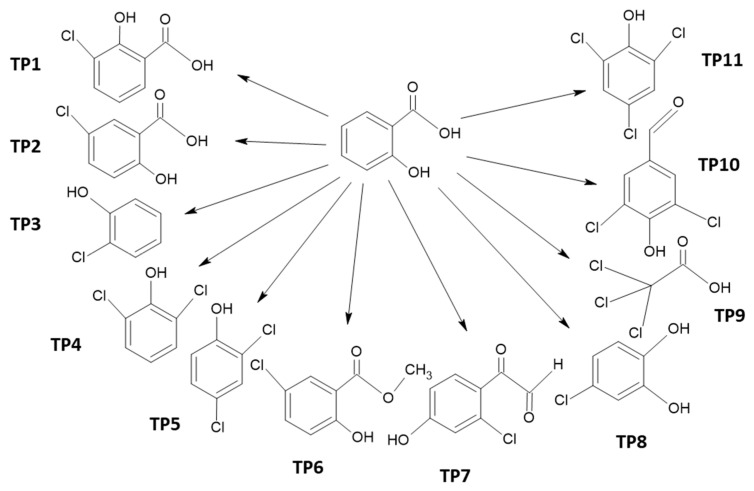
Scheme of transformation of main products of SA photodegradation: (TP1) 3-chloro-2-hydroxybenzoic acid, (TP2) 5-chloro-2-hydroxybenzoic acid, (TP3) 2-chlorophenol, (TP4) 2,6-dichlorophenol, (TP5) 2,4-dichlorophenol, (TP6) methyl 5-chlorosalicylate, (TP7) (2-chloro-4-hydroxyphenyl)(oxo)acetaldehyde, (TP8) 4-chlorobenzene-1,2-diol, (TP9) trichloroacetic acid, (TP10) 3,5-dichloro-4-hydroxybenzaldehyde, and (TP11) 2,4,6-trichlorophenol.

Based on the identified products, a scheme for the transformation of SA under the influence of NaOCl/UV was proposed (Figure 4).

### 2.3. Environmental Assessment of Chloroproducts of Salicylic Acid Intermediates

The identified intermediate products allow for the assessment of the potential environmental risk associated with the chlorination of SA using sodium hypochlorite and UV radiation. Table 3 presents the physicochemical properties and environmental characteristics of chlorinated products calculated using EPI SuiteTM 4.11 software [43].

**Figure 5:**
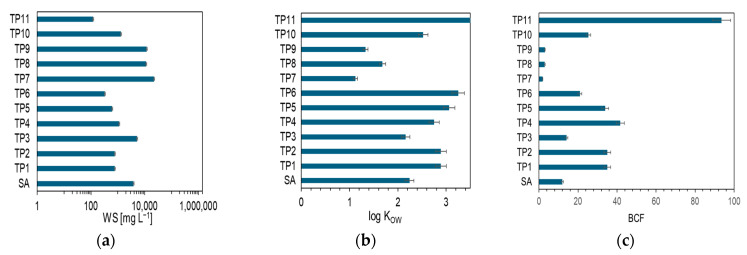
Selected environmentally relevant parameters calculated using EPI suite software: (WS) water solubility (**a**), (Log K_OW_) logarithmic value of n-octanol–water partition coefficient (**b**), (BCF) bioconcentration factor, and half-life values (**c**).

Boiling point (BP) and vapor pressure (VP) provide information on whether a compound will be emitted into the atmosphere relatively quickly after being released into the environment. Typically, an organic compound is considered volatile if it has 15 or fewer carbon atoms, its vapor pressure is greater than 10 Pa (0.75 mmHg) at 25 °C, and its boiling point at atmospheric pressure is less than 260 °C [44]. Table 3 shows that 2-chlorophenol (TP3), 2,6-dichlorophenol (TP4), and trichloroacetic acid (TP9) meet these criteria. The remaining chlorinated products have a VP below 7.5 × 10^−2^ mmHg and can therefore be classified as low-volatility compounds. The BP and VP parameters indicate that the organochlorine transformation products of SA do not tend to evaporate and remain in the liquid phase. The water solubility (WS) parameter suggests the fate of substances in water bodies. The solubility of SA in water at 25 °C is 3808 mg·L^−1^ (Figure 5a). Significantly higher solubility is exhibited by (2-chloro-4-hydroxyphenyl)(oxo)acetaldehyde (TP7); trichloroacetic acid (TP9); 4-chlorobenzene-1,2-diol (TP8); and 2-chlorophenol (TP3). 2,4-Dichlorophenol and 2,6-dichlorophenol have a high WS coefficient, but lower than SA. The ability of organochlorine substances to dissolve in water depends on the number of chlorine atoms present in the molecule structure and their position. An example of this is phenol, whose WS = 26,200 mg·L^−1^ [29].

The strongly electronegative chlorine atom reduces the electron density on the ^−^OH group, which reduces the molecule’s ability to form hydrogen bonds. The introduction of a single chlorine atom into the phenol molecule in the ortho-position reduces its solubility in water fivefold. Adding another atom and forming 2,6-dichlorophenol reduce solubility twentyfold, while 2,4-dichlorophenol reduces it more than thirtyfold. In the case of 2,4,6-trichlorophenol, solubility is lowest, at WS = 121 mg·L^−1^ (Table 3). It can be concluded that compounds that do not tend to evaporate and migrate into the air can migrate with water over considerable distances, undergo changes under the influence of environmental factors, or be adsorbed by aquatic organisms [45,46]. However, considering the Log K_OW_ and BCF parameters, it can be concluded that SA and virtually all chlorinated transformation products have a low potential to accumulate in living organisms (Log K_OW_ < 3 and BCF < 100) (Figure 5b,c). Only TP5, TP6, and TP11 can be classified as lipophilic compounds (Log K_OW_ > 3). Therefore, as a result of environmental contamination with SA and its intermediate products, it can be concluded that the water matrix will be the dominant one in which these compounds will migrate. This is confirmed by research conducted by scientists who found that chlorophenols used as disinfectants, pesticides, herbicides, and components in the production of medicines [47] or wood preservation [48] enter the environment and cause pollution. They have been identified in chlorinated tap water, groundwater and surface water resources [49], agricultural areas [50], and industrial wastewater [51]. According to data provided by the WHO, disinfection of tap water with chlorinating agents contributes to an increase in the concentration of chlorophenols [52]. The by-products of water treatment by chlorination are both 2,4-dichlorophenol and 2,5-dichlorophenol [53]. 2,4-Dichlorophenol was detected in more than half of surface water samples in China, with the highest concentration of 19.96 mg·L^−1^ [54]. The average concentration of 2,4,6-trichlorophenol in drinking water sources in three southwestern states of Nigeria ranged from 169 to 131 μg·L^−1^ [55].

In turn, in samples from the agricultural region of Boland in the Western Cape Province of South Africa, 4-chlorophenol and 2,4-dichlorophenol were identified at trace levels of 0.88 ng·L^−1^ and 0.17 µg·L^−1^, respectively [56]. The presence of trichlorophenol was also confirmed in sediments of Canadian streams [57].

**Figure 6:**
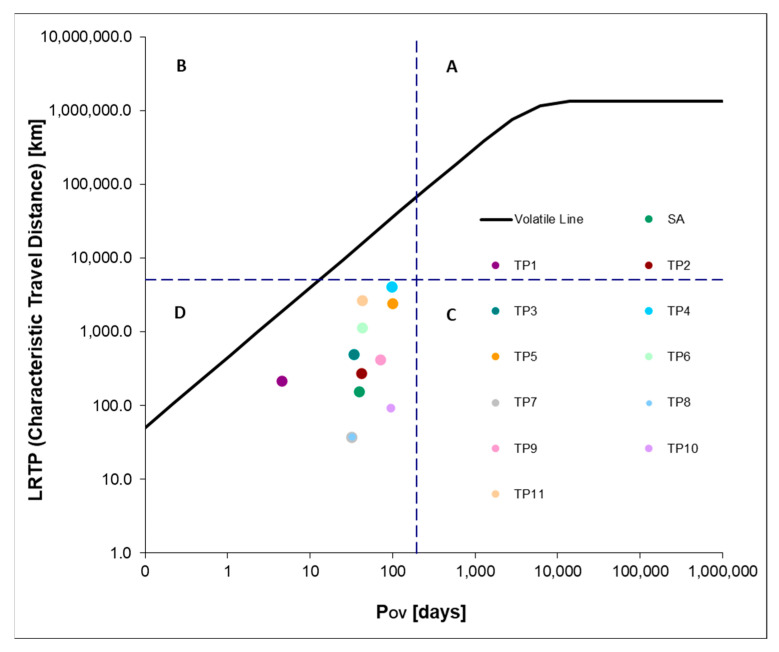
Long-range transport potential (LRTP) versus overall persistence (Pov) of SA and intermediates.

Based on Log K_AW_ and Log K_OW_ values and half-lives in air, water, and soil, both long-range transport potential (LRTP) and overall persistence in the environment (Pov) can be determined using the OECD POV and LRTP Screening Tool ver. 2.2 [58]. The LRTP indicator is described, among other things, by the characteristic travel distance (CTD) parameter, defined as the distance from the emission source to the point where the concentration of the substance falls to 38% of its initial value. According to the adopted methodology, the CTD parameter refers to the environment treated as a multimedia system, taking into account the migration of substances in air, water, and soil. The classification presented in Figure 6 is based on the approach described by Klasmeier et al. [59], in which chemicals are grouped into categories using a set of reference compounds. These include typical persistent organic pollutants such as pesticides and polychlorinated biphenyls. The boundaries between categories were established based on CTD and Pov thresholds, defining four areas (Figure 6). The group of compounds with high mobility and persistence includes those with a CTD above 5096.73 km and a Pov exceeding 195 days. According to this approach, SA and all identified products of its photodegradation show low transport potential in the environment. However, it is worth noting TP4 (96.82 days) and TP5 (99.62 days), which are characterized by the highest environmental persistence among the compounds studied and the highest LRTP values: TP4 (4115.10 km) and TP5 (2473.19 km). Although the results obtained classify the compounds studied into Region D, they indicate that they may be capable of long-range transport and persistence in the environment, which potentially makes them a threat to the environment.

### 2.4. Toxicological Evaluation of Chloroproducts of Salicylic Acid Transformation

**Figure 7:**
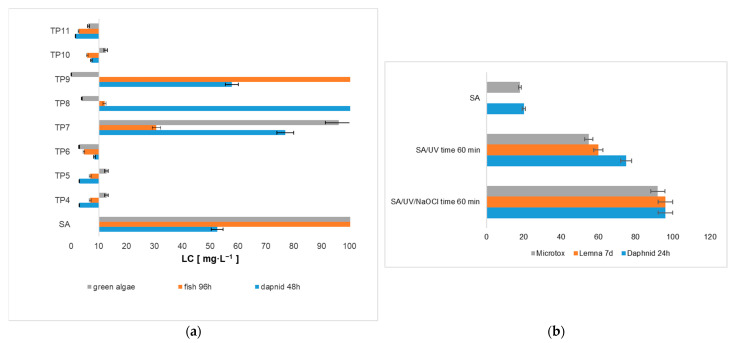
Toxicity of SA and its chlorinated transformation products: toxicity results calculated using Ecosar (**a**); toxicity results using biotests (**b**).

Information on the toxicity of SA transformation products is scarce. This gap is likely due to the inherent difficulties in capturing all intermediate degradation steps. In addition, difficulties in isolating degradation products or the lack of reference standards for detected compounds further complicates toxicological studies. Therefore, mathematical models based on the structural characteristics of detected products are increasingly being used for the ecotoxicological assessment of emerging pollutants [60]. This approach allows for the rapid acquisition of preliminary data, reduces the costs of experimental research, and directs the attention of researchers to substances with the greatest potential risk to the environment. Computational approaches are becoming increasingly valuable, and such analyses are now standard practice [60,61,62]. Based on the 96 h LC_50_ results for fish calculated using ECOSAR ver. 2.2., nearly half of the identified chlorinated SA degradation products exhibit acute toxicity, with LC_50_ values below 10 ppm (Figure 7a). These include TP4, TP5, TP6, TP10, and TP11. In a 48 h LC50 test on daphnia (In the Daphnid), TP4, TP5, TP10, and TP11 showed significant toxicity, while TP6 showed no acute toxic effects. Finally, in the 96 h EC_50_ test on green algae, only TP6, TP8, and TP11 were identified as particularly toxic. According to the calculated LC_50_ values from the 96 h test on fish and the 48 h test on daphnia (In the Daphnid), TP11 showed the highest toxicity. Computational analyses also indicate that TP6 is the most toxic compound to green algae, with TP9 ranking second. Chlorophenols are an important group of environmental pollutants that are of concern due to their toxicity and widespread use. Of the 11 SA transformation chloroproducts, 2,4-dichlorophenol and 2,4,6-trichlorophenol are on the US Environmental Protection Agency’s Priority Pollutants List [63]. One of the most toxic chlorophenols on the list is 2,4-dichlorophenol. The short phenoxy side chain and low steric hindrance of the compound, and above all its lipophilic nature, facilitate its penetration through cell membranes, thereby increasing its toxic effect. It has been shown that after a single intravenous injection of 10 mg/kg in male Sprague Dawley (SD) rats, 2,4-dichlorophenol was rapidly distributed to the kidneys, liver, brain, adipose tissue, and plasma of the rats [64].

Research suggests that exposure of animals and humans to dichlorophenol increases the incidence of asthma. It also contributes to an increase in the incidence of food allergies [47]. Low concentrations of 2,4-dichlorophenol caused developmental disorders in zebrafish embryos and showed weak reproductive toxicity in rats [54]. In the case of 2,4,6-trichlorophenol, there is evidence confirming the carcinogenic properties of the compound. The presence of trichlorophenol in the diet of animals used for research caused leukemia in rats and also induced liver tumors in mice [65]. The toxic effects of 2,4,6-trichlorophenol are also confirmed by studies on the catfish *Clarias batrachus*. The results showed that even at low concentrations and short exposure times, trichlorophenol significantly impaired the physiological parameters of the fish (hematological, biochemical, growth, and reproduction) [57]. It is known that the position of C-Cl in relation to the ^−^OH group strongly activates the aromatic ring, and the presence of chlorine atoms affects the electronic properties of the ring [66]. Such a molecule is chemically stable, and due to its lipophilic nature, it easily penetrates cell membranes and interacts with organic matter [67]. Based on the available data on the chemical structure of the compound and its impact on living organisms, the United Nations Environment Programme (UNEP) has classified 2,4,6-trichlorophenol as a persistent organic pollutant subject to legal restrictions [68]. In the case of 2,6-dichlorophenol, it has been confirmed that it inhibits the process of spore production in fungi and is toxic to microorganisms involved in the biodegradation of organic matter [69,70]. In the case of rats, the acute toxicity, LD 50, is 390 mg·kg^−1^, while the LD 50 for mice is 2120 mg kg^−1^, with minimal toxicity after a single ingestion. However, there are no recent studies in the literature showing direct health effects in humans. In the case of methyl-5-chlorosalicylate, there are no data available on the toxicity of the compound. There is data regarding methyl salicylate, which is toxic to humans. Methyl salicylate is a relatively common cause of poisoning in children, and ingestion of one teaspoon of wintergreen oil can be fatal, as it contains about 6 g of salicylate, which is equivalent to ingestion of about 20 aspirin tablets [71].

In order to evaluate the effectiveness of oxidation processes, it is necessary to assess and compare their potential biological and toxicological effects. Therefore, the authors evaluated the toxicity of reaction mixtures using three in vitro biotests: Microtox^®^, Daphtoxkit F^®^, and the *Lemna* sp. Microtox^®^ test. The selected tests offer a rapid method for detecting acute toxicity, making them valuable for identifying harmful by-products at low concentrations [72]. These tests are widely used to evaluate various contaminants, including pharmaceutical products, and provide reliable data for risk assessment [72,73,74]. It is worth noting that biotests are a good supplement to the calculated toxicological values for individual transformation products. Toxicological studies make it possible to test real samples with transformation products, which may interact with each other and cause a synergistic or inhibitory effect in terms of toxicity to living organisms. To assess the differences in the toxicity of the mixture after the chlorination process involving UV radiation, the toxicity was compared with the initial SA solution and with the mixture subjected only to UV (Figure 7b). The tested systems showed higher toxicity compared to the unprepared SA solution. Control samples containing all reagents except SA showed minimal toxicity, indicating that the observed increase in toxicity is mainly due to the action of specific transformation products rather than the factors used in the study themselves. It is worth noting that the post-reaction mixture from the NaOCl/UV system was more toxic than the post-UV solution. This confirmed the predicted toxicity to aquatic organisms, determined on the basis of theoretical calculations, which classified most chlorinated transformation products of SA as toxic.

## 3. Materials and Methods

### 3.1. Reagents

Analytical standards of SA with analyticalpurity were purchased from Sigma Aldrich (Saint Louis, MO, USA). Sodium hypochlorite (NaOCl) (10%) was purchased from POCh (Gliwice, Poland). Methanol, ethyl acetate, water, and formic acid used in chromatographic analyses were purchased from Sigma Aldrich. Test kits were used to assess the toxicity of Daphtoxkit F^®^ and *Lemna* sp. Reagents for the GIT (growth inhibition test) and Microtox^®^ biotest were purchased from TIGRET z.o.o. (Warsaw, Poland).

### 3.2. Experiments on the Photodegradation of Salicylic Acid and Its Transformation Products

Photodegradation experiments were carried out in a photoreactor (Heraeus, Hanau, Germany) equipped with a medium-pressure mercury lamp (TQ150W, Heraeus, Hanau, Germany), cooled with tap water to a temperature of 20 ± 1 °C. The photoreactor was placed on a magnetic stirrer to ensure the homogeneity of the reaction mixture throughout the entire volume. The radiation source was polychromatic light, whose characteristics were determined based on the following excitation wavelengths: 313, 365, 405, 436, 546, and 578 nm. The corresponding radiation intensities were 2.5, 5.8, 2.9, 3.6, 4.6, and 4.2 W, respectively.

Aqueous solutions of SA with an initial concentration of 0.36 mM were used for the tests. Changes in concentration were monitored by liquid chromatography with a DAD detector at a measurement wavelength of 300 nm. The effect of pH on the photodegradation of salicylic acid, SA, in the presence of NaOCl (1 mM) was studied in reaction systems adjusted to pH values of 3.5, 7.5, and 10. The effect of NaOCl concentration on the photodegradation rate was evaluated for concentrations of 0.4 mM, 1 mM, and 2 mM, at a constant pH = 7.5. To maintain specific pH conditions, buffer solutions (0.1 M) were prepared: citrate buffer for pH 3.5, and phosphate buffers for pH 7.5 and 10. Subsequently, 1 L of SA solution was prepared in a volumetric flask for each photodegradation condition by dissolving SA in the respective buffers. For photodegradation measurements, 0.7 L of the solution was introduced into the photoreactor.

### 3.3. Detection of Salicylic Acid Transformation Products

The reaction mixtures were analyzed using a gas chromatograph with the GC–MS 5890 HEWLETT PACKARD mass spectrometry detector (Palo Alto, CA, USA) and ZB-5MS column (0.25 mm × 30 m × 0.25 μm). Analyses were performed under the following chromatographic operating conditions: injector temperature of 250 °C, detector temperature of 280 °C, and oven temperature program from 80 to 300 °C (kept for 2 min) at 10 °C/min. Helium was used as a carrier gas. The sample volume was 1 μL. Reaction products were identified by comparing MS spectra obtained with those of the NIST/EPA/NIH mass spectrum library.

A Shimadzu UFLC XR liquid chromatograph equipped with DAD and MS detectors (LC-MS 2020 Shimadzu, Kyoto, Kyoto Prefecture, Japan) was used to detect the transformation products. A Phenomenex Kinetex C18 column (3 mm × 100 mm, 2.6 μm) (Torrance, CA, USA) was used. The mobile phase consisted of polar component A (water with 0.1% HCOOH) and less polar component B, which was methanol. The following chromatographic conditions were used: flow rate of 0.4 mL·min^−1^, column temperature of 45 °C, and injection volume of 5 μL. The detected transformation products were analyzed using a 2020 Shimadzu LC-MS system equipped with an electrospray ionization source. The negative ion *m*/*z* 138 was selected to monitor SA. In addition, the entire range of 50–750 *m*/*z* was scanned in positive and negative ionization to detect the presence of other analytical signals. The operating conditions of the mass spectrometer were as follows: drying gas flow rate (N_2_) of 1.5 mL·min^−1^, gas temperature of 400 °C, nebulizer gas pressure of 30 psi, fragmentor voltage of 70 V, and capillary voltage of 4000 V. Details of the chromatographic separation are given in a previous article [29].

### 3.4. Determination of Physicochemical and Ecotoxicological Parameters of Intermediate Compounds

The EPI Suite™ 4.11 [43] package, which is based on QSAR (quantitative structure–activity relationship) methods, was used to predict the physicochemical properties of SA and its intermediate products. These models enabled the estimation of a number of key indicators, including melting point (MP) and boiling point (BP), water solubility (WS), vapor pressure (VP), bioconcentration factor (BCF), and logarithms of partition coefficients: octanol/water (Log K_OW_), octanol/air (Log K_OA_), organic carbon/water (Log K_OC_), and air/water (Log K_AW_). In addition, Henry’s constant (KH) and acute toxicity parameters LC_50_ and EC_50_ were calculated. For all SA transformation products, POV and LRTP values were also determined using the dedicated POV and LRTP Screening Tool [75].

### 3.5. Toxicity Assessment of Intermediate Compounds

The toxicological effects of SA chlorination products were determined in accordance with the procedures described in previous studies [45,46]. A 0.36 mM SA solution in deionized water was subjected to UV and NaOCl/UV treatment, carried out after the photooxidation process (60 min). Samples were taken before and after each process, then diluted in a 1:100 ratio, and analyzed for toxicity. At the same time, control samples were prepared according to the same procedure, but without the addition of SA, in order to exclude the influence of factors unrelated to the presence of the tested compound. The results for the systems containing SA were compared with the results of the control samples, which allowed the toxicity of the resulting degradation products to be determined. In all control tests, a very low toxicological effect was observed, not exceeding 2%. Each variant of the experiment was performed in four replicates, which ensured the repeatability and reliability of the data.

Three independent biological tests were used to assess toxicity: Microtox^®^, Daphtoxkit F^®^, and the *Lemna* sp. growth inhibition test (GIT). The Microtox^®^ test, performed using a Microtox Model 500 analyzer (Modern Water, Warsaw, Poland) and the MicrotoxOmni protocol, measured the degree of inhibition of bioluminescence of *Aliivibrio fischeri* bacteria after 5 min of exposure to the test samples, and the values obtained were presented as a percentage of inhibition relative to the control. The second test—Daphtoxkit F^®^—was based on the indicator organism Daphnia magna and was performed in accordance with the requirements of ISO 6341 [76] and OECD 202 [77,78]. For the *Lemna* sp. GIT, plants grown under laboratory conditions were maintained at a temperature of 25 ± 1 °C under constant illumination of 6000 lx. After 7 days of exposure, morphological changes and the degree of leaf growth inhibition were assessed. The procedure was in accordance with OECD Guideline No. 221, commonly used in the assessment of the toxicity of new pollutants, including pharmaceutical substances [79].

### 3.6. Data Analysis and Replication

All experimental measurements were performed in four independent replicates for each condition. The reported results represent the arithmetic mean of these replicates. The standard deviation (SD) was calculated for each dataset to estimate variability. In all cases, the calculated standard deviations did not exceed 5% of the mean value, indicating high reproducibility. Error bars representing ±1 SD are included on figures, providing a visual indication of experimental variability. This approach ensures that the reported values accurately reflect the central tendency of the measurements while clearly acknowledging the minimal experimental uncertainty.

## 4. Conclusions

The conducted studies on the photooxidation of salicylic acid (SA) in the presence of sodium hypochlorite (NaOCl) demonstrated that NaOCl alone is only moderately effective in SA degradation, due to the low oxidizing potential of the OCl^−^ species prevailing under alkaline conditions. The application of UV radiation in combination with NaOCl significantly enhanced the decomposition rate of SA, especially under neutral and alkaline pH. However, this process was accompanied by the formation of numerous chlorinated transformation products, including chlorophenols, chlorobenzoic acids, chlorinated aldehydes, and trichloroacetic acid, among others. Specifically, the following products were identified: 3-chloro-2-hydroxybenzoic acid; 5-chloro-2-hydroxybenzoic acid; 2-chlorophenol; 2,6-dichlorophenol; 2,4-dichlorophenol; methyl 5-chlorosalicylate; (2-chloro-4-hydroxyphenyl)(oxo)acetaldehyde; 4-chlorobenzene-1,2-diol; trichloroacetic acid; 3,5-dichloro-4-hydroxybenzaldehyde; and 2,4,6-trichlorophenol. It should be stressed that product identification was carried out without analytical standards, and thus their occurrence and quantitative relevance require confirmation in further studies.

Ecotoxicological testing and QSAR model calculations confirmed that many of the chlorinated intermediates exhibit considerable toxicity towards fish, daphnia, and algae. Moreover, the reaction mixture obtained after the NaOCl/UV process displayed higher toxicity than that resulting from direct SA photolysis, demonstrating that UV-assisted chlorination, although effective in accelerating SA degradation, simultaneously generates transformation products of greater persistence and ecological impact.

These findings highlight the necessity of future investigations aimed at monitoring chlorinated transformation products of pharmaceuticals in real environmental samples, where complex matrices such as dissolved organic matter as well as selected anions and cations may influence degradation pathways and product profiles. It also appears crucial to develop purification approaches that minimize the formation of persistent and toxic by-products, for example, by employing alternative oxidants, adjusting reaction conditions, or integrating photocatalytic steps with biological treatment. Additionally, the analysis of synergistic and antagonistic interactions within mixtures of transformation products should be pursued, as such effects can substantially alter overall toxicity.

In conclusion, the research has shown that while UV-assisted NaOCl photooxidation substantially improves the degradation efficiency of salicylic acid, it also results in the formation of chlorinated products with enhanced environmental persistence and toxicity, raising concerns about their potential impact on aquatic ecosystems.

## Figures and Tables

**Table 1:** The main products of SA photodegradation assisted by NaOCl/UV identified using LC-MS.

Retention Time, T_R_	Compound	Molecular Weight	[M + H]^+^
8.45	4-chlorobenzene-1,2-diol	144.50	145
11.17	(2-chloro-4-hydroxyphenyl)(oxo)acetaldehyde	185.63	186
10.91	trichloroacetic acid	163.00	164
1.02	3,5-dichloro-4-hydroxybenzaldehyde	191.00	192
15.80	5-chloro-2-hydroxybenzoic acid	172.56	173
15.80	3-chloro-2-hydroxybenzoic acid	172.56	173
11.60	2,4,6-trichlorophenol	197.50	198
10.85	methyl 5-chlorosalicylate	186.59	187

**Table 2:** The main products of SA photodegradation assisted by NaOCl/UV identified using GC-MS.

Retention Time, T_R_	Compound	Molecular Weight	*m*/*z*
8.456	2-chlorophenol	128.55	64, 92, 128, 130
10.682	2,6-dichlorophenol	163.00	63, 98, 126, 162, 164
11.063	2,4-dichlorophenol	163.00	63, 98, 162, 164
13.997	5-chloro-2-hydroxybenzoic acid	172.56	63, 126, 154, 172
14.347	3-chloro-2-hydroxybenzoic acid	172.56	154, 156, 172
16.598	methyl 5-chlorosalicylate	186.59	63, 126, 154, 186

**Table 3:** Physicochemical parameters and environmental characteristics of SA and its chlorination products.

Compound	No. TP	BP, °C	MP, °C	VP, mmHg	WS, mg/L	Henry’s LC	Log K_OW_	Log K_AW_	Log K_OA_	Log K_OC_	BCF
SA		298	94	8.2 × 10^−5^	3808	1.5 × 10^−9^	2.24	−6.5	8.8	1.6	11.9
3-chloro-2-hydroxybenzoic acid	TP1	320.7	114.4	2.6 × 10^−5^	775	1.1 × 10^−8^	2.89	−6.4	9.3	1.9	34.9
5-chloro-2-hydroxybenzoic acid	TP2	320.7	114.4	2.6 × 10^−5^	775	1.1 × 10^−8^	2.89	−6.4	9.3	1.9	34.9
2-chlorophenol	TP3	203.1	28.6	95.5	5165	1.2	2.16	−3.3	5.5	2.3	14.1
2,6-dichlorophenol	TP4	233.7	46.8	3.02	1130	3.1 × 10^−7^	2.75	−3.9	6.7	2.6	41.7
2,4-dichlorophenol	TP5	233.7	46.8	0.0657	614.2	3.1 × 10^−7^	3.06	−3.8	6.8	2.8	33.9
Methyl 5-chlorosalicylate	TP6	279	69.4	0.00128	327	3.4 × 10^−6^	3.25	−3.9	7.1	2.8	20.9
(2-chloro-4-hydroxyphenyl)(oxo)acetaldehyde	TP7	312	96.3	6.5 × 10^−5^	2.2 × 10^4^	4.1 × 10^−12^	1.12	−9.8	10.9	1	1.9
4-chlorobenzene-1,2-diol	TP8	258.4	69.9	7.3 × 10^−4^	1.1 × 10^4^	4.3 × 10^−11^	1.68	−8.8	10.4	2.2	2.9
trichloroacetic acid	TP9	203.2	26.7	0.75	1.2 × 10^4^	1.4 × 10^−8^	1.3	−6.3	7.6	0.5	3.0
3,5-dichloro-4-hydroxybenzaldehyde	TP10	292.8	83.1	2.8 × 10^−4^	1.3 × 10^3^	7.7 × 10^−10^	2.5	−7.5	10.0	1.6	25.3
2,4,6-trichlorophenol	TP11	262.1	63.8	5.4 × 10^−3^	1.2 × 10^2^	2.6 × 10^−6^	3.6	−3.9	7.6	3.3	93.6

(BP) Boiling point, (MP) melting point, (VP) vapor pressure, (WS) water solubility, (Henry’s LC) Henry’s law constant, (log K_OW_) logarithmic value of n-octanol–water partition coefficient, (log K_AW_) logarithmic value of air/water partition coefficient, (log K_OA_) logarithmic value of n-octanol/air partition coefficient, (log K_OC_) logarithmic value of organic carbon/water partition coefficient, (BCF) bioconcentration factor, and half-life values.

## Data Availability

The data presented in this study are available upon request from the corresponding author.
